# Targeting an Ischemic Time <120 Minutes in ST‐Segment–Elevation Myocardial Infarction

**DOI:** 10.1161/JAHA.119.013067

**Published:** 2019-06-11

**Authors:** Matthew Henderson, Jaclyn Carberry, Colin Berry

**Affiliations:** ^1^ University of Glasgow United Kingdom

**Keywords:** Editorials, door‐to‐balloon, fibrinolysis, myocardial infarction, primary angioplasty, symptom‐to‐balloon, Myocardial Infarction, Mortality/Survival, Magnetic Resonance Imaging (MRI), Catheter-Based Coronary and Valvular Interventions

## Introduction

Despite advances in the treatment of ST‐segment–elevation myocardial infarction (STEMI), failed myocardial reperfusion (microvascular obstruction) occurs in half of STEMI patients,^1^, ^2^, ^3^, ^4^ and is independently predictive of all‐cause death and heart failure in the longer term.^4^ On this basis, we contend that primary percutaneous coronary intervention (PCI) should not be classified as successful when myocardial reperfusion has failed. The improvements in early survival during acute STEMI that have been achieved through advances in emergency care shift the healthcare burden downstream, growing the population of survivors with injured hearts (ie, microvascular obstruction) who are at risk of heart failure in the longer term.

A key factor in the treatment of STEMI is the ischemic time, in other words, the time from symptom onset to therapeutic reperfusion of infarcted myocardium.^2^ The longer the artery is occluded, the more the wavefront of ischemia extends radially from the endocardium to the epicardium.^3^ Importantly, the transmural extent of infarction and overall size of infarction is independently predictive of cardiac prognosis in the longer term.^5^ In this regard, cardiac magnetic resonance imaging (CMR) is a powerful prognostic tool for the assessment of myocardial pathology and function, enabling risk stratification of STEMI patients^4^, ^6^ (Figure).

**Figure 1:**
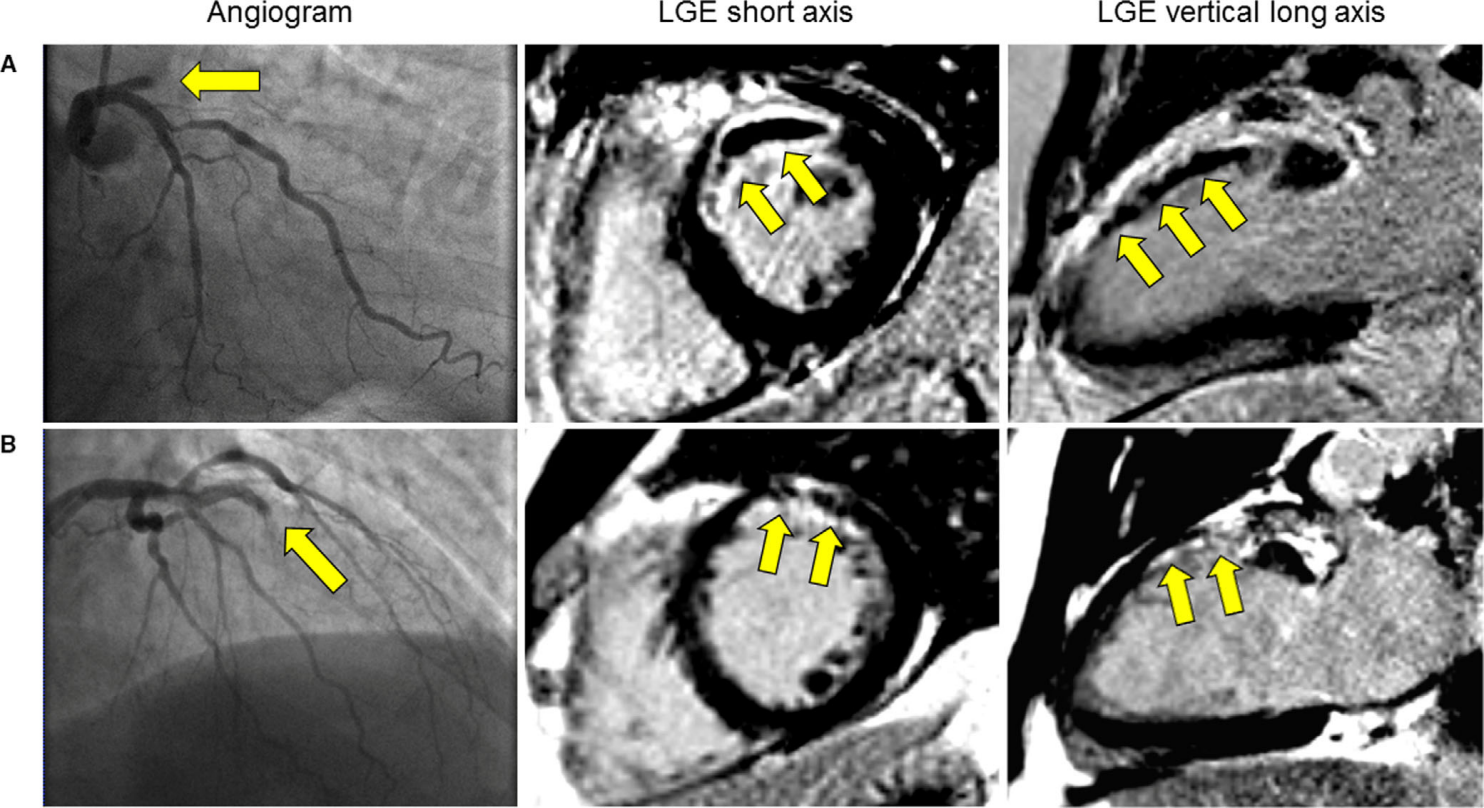
Two patients with similar presentations of STEMI. Both patients had Thrombolysis in Myocardial Infarction 0 flow in the left anterior descending artery (yellow arrows), with Rentrop 0 collateral flow. Both patients were treated according to guidelines. **A**, Patient with transmural infarct and microvascular obstruction. Symptom‐to‐balloon time was 163 minutes. LGE imaging demonstrated infarct size of 25% of the left ventricle. Microvascular obstruction was present at 8% of the left ventricle. Myocardial salvage index was 30%. This patient also had myocardial hemorrhage acutely that persisted as myocardial iron at a 6‐month follow‐up CMR (not shown). This patient was rehospitalized with new‐onset heart failure. **B**, Patient without transmural infarct and no microvascular obstruction. Symptom‐to‐balloon time was 87 minutes. LGE imaging demonstrated infarct size of 16% of the left ventricle. Myocardial salvage index was 52%. This patient had an uncomplicated clinical course. CMR indicates cardiac magnetic resonance; LGE, late gadolinium enhancement; STEMI, ST‐segment–elevation myocardial infarction.

In this issue of the *Journal of the American Heart Association* (*JAHA*), Greulich and colleagues report a multicenter study of 374 screened patients presenting with acute STEMI.^7^ A subset of 164 patients (mean age 54 years, 80% male) presenting with a first‐time STEMI within 12 hours of symptom onset, an occluded culprit coronary artery (Thrombolysis in Myocardial Infarction flow 0), single‐vessel disease, and no coronary collaterals (Rentrop 0) were included in a prospective CMR study. Infarct extent and distribution were assessed using late gadolinium enhancement imaging. The transmurality index was defined as 100‐myocardial salvage index. Transmurality grade (<25%, 25–50%, 51–75%, and 76–100%) was visually graded, with a transmurality grade of 76% to 100% indicating a transmural infarct. The main findings were that patients with a symptom‐to‐balloon time of >121 minutes had a significantly higher transmurality index and grade, larger infarct size, and decreased myocardial salvage index. More patients with a symptom‐to‐balloon time >121 minutes had transmural infarction (96% versus 64%; *P*<0.001). These observations extend the pathological evidence underpinning the clinical importance of limiting the symptom‐to‐balloon time.^8^, ^9^

Placing the results of this study in a wider context,^7^ the observed symptom‐to‐balloon time (133 [103–196] minutes^7^) was shorter than the ischemic time (174 [120, 311] minutes) of an all‐comers cohort with STEMI treated in the West of Scotland Optimal Reperfusion Service,^4^ a regional care network. This difference likely reflects a combination of factors, including geographic and socioeconomic differences. Despite the shorter ischemic time, a comparatively high proportion of patients in Greulich's cohort had microvascular obstruction (64%) compared with previous studies.^7^ This is likely explained by selection criteria applied to the analysis population. Importantly, microvascular obstruction was more common and extensive in patients with a symptom‐to‐balloon time >121 minutes, which is consistent with the literature.^2^ The high prevalence of microvascular obstruction, its causal association with myocardial hemorrhage,^6^ and lack of any effective therapy presents a major, unmet need.^10^ As a case in point, in the T‐Time (Trial of Low‐dose Adjunctive alTeplase During prIMary PCI), the lack of efficacy of low‐dose intracoronary alteplase, as compared with placebo, when given during primary PCI after reperfusion and before stenting, highlights the need for more research.^11^

The authors provided a focused analysis on the transmural extent of infarction in relation to ischemic time. In a subset of 15 patients with <50% transmurality, 3 patients who had no evidence of myocardial necrosis by CMR had a mean symptom‐to‐balloon time of 56±15 minutes. When all 12 patients were assessed, the mean symptom‐to‐balloon time was increased to 77±29 minutes. Clearly, the time margins for reperfusion of viable myocardium are small, reinforcing the case for reducing the time from symptom onset to reperfusion.

Greulich et al^7^ also observe that infarct size is somewhat confounded by the infarct‐related artery. An occluded left circumflex artery with symptom‐to‐balloon time of >121 minutes and higher transmural extent of infarction could culminate in a smaller infarct size than would be the case following occlusion of the left anterior descending coronary artery for ≤121 minutes and less transmurality. So, can transmurality provide any prognostic benefit over the infarct size? Ahn et al^5^ found that the transmural necrotic segment count is more predictive of left ventricular remodeling and clinical outcome than the infarct size in a post‐STEMI population.

The selection criteria applied by Greulich et al^7^ enabled the research question to be efficiently addressed within a comparatively homogeneous subgroup of patients. Confounding factors, such as variations in antegrade flow in the culprit artery, collateral blood supply, and multivessel disease, are mitigated out. Their findings have important clinical implications that are relevant to practice guidelines for STEMI. Current guidelines recommend primary PCI in patients with a symptom‐to‐balloon time of up to 12 hours, provided intervention can be delivered within 120 minutes of “STEMI diagnosis.”^1^ The message emphasizes the therapeutic priority for implementation of prehospital care networks that can efficiently diagnose STEMI in the community and rapidly transfer the patient to regional primary PCI centers, and when the first medical contact to PCI is >120 minutes, then lytic therapy can be administered prehospital followed by direct transfer to the PCI center, circumventing the emergency department.^12^ Public education is key to inform patients and the public on how to recognize the symptoms of acute STEMI, thereby reducing the time from symptom onset to the call for help. This message is especially relevant to women, who may not recognize the symptoms as being ischemic in origin, who may be less well placed to call for help, and when they do call for help, may be less likely to be positively diagnosed by first responders and emergency care (male) clinicians. Public health interventions represent key areas for future research and advocacy. Reflecting the importance of the efficiency of medical care, door‐to‐balloon time has been identified as being more closely associated with 1‐year mortality than symptom onset‐to‐door time.^9^ In patients with a delayed hospital presentation beyond 12 hours, factors relating to the patient's decision to seek help are a main component of delay.^13^ This strengthens the argument for using patient education to improve STEMI outcomes.

The study by Greulich et al^7^ has a few limitations. First, CMR was performed a median of 4 days postreperfusion, which is a little early to assess infarct size post‐STEMI, and the interquartile range of 2 to 6 days was rather broad. Nonetheless, this timescale reflects real‐life clinical practice. In a time‐course study with CMR performed at 4 to 12 hours‐, 3‐, and 10 days post‐STEMI, infarct size and myocardial salvage index were differentially associated with the timing point post‐MI.^6^

Second, the CMR studies only used late gadolinium enhancement, and contemporary CMR mapping techniques such as T1, T2, or T2* are not available. Tissue characterization to assess remote zone inflammation, myocardial hemorrhage, and edema in relation to the symptom‐to‐balloon time would have been of interest.

Third, reporting bias is an inherent limitation of studying symptom‐to‐balloon time. Patients may find it difficult to accurately estimate the time of symptom onset, especially in the acute setting when suffering from pain and stress. Therefore, symptom‐to‐balloon times may not be independently verified. Door‐to‐balloon times can be more reliably and efficiently collected, which may in part account for the prominence of clinical evidence in this regard, as well, of course, as the importance of measuring performance in emergency care.

In conclusion, the study by Greulich et al^7^ provides evidence of associations between a prolonged symptom onset to balloon time >121 minutes and increasing transmural extent of infarction and infarct size. The findings highlight the limited therapeutic potential for myocardial salvage in patients presenting >120 minutes even when <12 hours. Shorter symptom‐to‐balloon times are vital to limit myocardial damage and increase myocardial salvage. When timely primary PCI is not feasible, lytic therapy before transfer to a PCI‐capable hospital should be considered, notably for patients presenting within 3 hours and unable to undergo primary PCI within 1 hour.^14^ Future studies should assess the prognostic impact of public health initiatives (eg, education) and interventions to improve the efficiency of interdisciplinary prehospital care networks.

## Sources of Funding

This work was supported by the British Heart Foundation (BHF) (PG/14/64/31043; RE/18/6/34217).

## Disclosures

Colin Berry is employed by the University of Glasgow, which holds consultancy and research agreements with companies that have commercial interests in the diagnosis and treatment of ischemic heart disease. The companies include Abbott Vascular, AstraZeneca, Boehringer Ingelheim, Heartflow, Philips, and Siemens Healthcare. These companies had no involvement in the current research or the manuscript. The remaining authors have no disclosures to report.
